# Antimicrobial resistance among* Streptococcus equi* subspecies *zooepidemicus* and *Rhodococcus equi* isolated from equine specimens submitted to a diagnostic laboratory in Kentucky, USA

**DOI:** 10.7717/peerj.13682

**Published:** 2022-09-21

**Authors:** Jennifer Lord, Craig Carter, Jacqueline Smith, Stephan Locke, Erica Phillips, Agricola Odoi

**Affiliations:** 1 Biomedical and Diagnostic Sciences, University of Tennessee, Knoxville, TN, United States of America; 2 Veterinary Diagnostic Laboratory, University of Kentucky, Lexington, KY, United States of America

**Keywords:** Logistic Regression, Antimicrobial Resistance, AMR, Multidrug Resistance, MDR, *Rhodococcus equi*, *Streptococcus equi*, Horse, Equine, Kentucky

## Abstract

**Background:**

Surveillance of antimicrobial resistance (AMR) among veterinary pathogens is necessary to identify clinically relevant patterns of AMR and to inform antimicrobial use practices. *Streptococcus equi* subsp.* zooepidemicus* and *Rhodococcus equi* are bacterial pathogens of major clinical importance in horses and are frequently implicated in respiratory tract infections. The objectives of this study were to describe antimicrobial resistance patterns and identify predictors of AMR and multidrug resistance (MDR) (resistance to three or more antimicrobial classes) among equine *S*.* zooepidemicus* and *R. equi* isolates.

**Methods:**

Antimicrobial susceptibility data from equine specimens submitted to the University of Kentucky Veterinary Diagnostic Laboratory between 2012 and 2017 were used in the study. Temporal trends in AMR and MDR were assessed using the Cochran-Armitage test. Logistic regression was used to identify associations between patient characteristics and the following outcomes: (a) MDR among *S. zooepidemicus* isolates*,* and (b) resistance to macrolides and ansamycins (rifampin) among *R. equi* isolates. Logistic regression was also used to investigate whether resistance of *S. zooepidemicus* and *R. equi* isolates to an antimicrobial class could be predicted by resistance to other drug classes.

**Results:**

The vast majority of *S. zooepidemicus* (99.6%) and *R. equi* isolates (83%) were resistant to at least one antimicrobial agent, but no significant temporal trends in AMR were observed. Approximately half (53.3%) of the *S. zooepidemicus* isolates were multidrug-resistant, and there was a significant (*p* < 0.001) increasing temporal trend of MDR among *S. zooepidemicus* isolates. Resistance to penicillin, which is typically recommended for treatment of suspected *S. zooepidemicus* infections, also increased during the study period, from 3.3% to 9.5%. Among *R. equi* isolates, 19.2% were resistant to one or more macrolide antibiotics, 24% were resistant to rifampin, and 15.6% were resistant to both macrolide(s) and rifampin. For both organisms, resistance to an antimicrobial class could be predicted based on resistance profiles to other drug classes. For instance, significant (*p* < 0.01) predictors of *β*-lactam resistance among *S. zooepidemicus* isolates included resistance to macrolides (Odds Ratio (OR) = 14.7) and ansamycins (OR = 9.3). Resistance to phenicols (OR = 3.7) and ansamycins (OR = 19.9) were associated with higher odds of macrolide resistance among *R. equi* isolates.

**Conclusions:**

The increase in MDR among *S. zooepidemicus* isolates is concerning. The observed levels of resistance to macrolides and rifampin among *R. equi* are also worrisome given the limited number of antimicrobials available for treatment of this organism. The findings of this study highlight the importance of ongoing surveillance of AMR to guide treatment decisions and directions for future research.

## Introduction

*Streptococcus equi* subspecies *zooepidemicus* and *Rhodococcus equi* are bacterial pathogens of horses associated with significant clinical and economic impacts. *S*. *zooepidemicus* is a commensal organism of the oral cavity, pharynx, and respiratory tract, and is frequently implicated as the cause of opportunistic respiratory infections in both foals and adults (Schroeder, 2014). In addition, *S. zooepidemicus* causes infections in other domestic species, and virulent *S. zooepidemicus* strains have been implicated in several recent high-mortality disease outbreaks in swine in North America (Sitthicharoenchai et al., 2020; De Costa & Lage, 2020; Chen et al., 2020). Human infections resulting from zoonotic transmission from contact with horses, dogs, and guinea pigs (Abbott et al., 2010; Minces, Brown & Veldkamp, 2011; Pelkonen et al., 2013; Gruszynski et al., 2015; Kittang et al., 2017; Kim et al., 2022), or from ingestion of uncooked meat or unpasteurized milk products (Kuusi et al., 2006; Kerdsin et al., 2021), have also been documented. Several studies have reported most or all equine *S. zooepidemicus* isolates to be susceptible to penicillins (Erol et al., 2012; Johns & Adams, 2015; Malo et al., 2016; Awosile et al., 2018), which remain the treatment of choice for suspected *S. zooepidemicus* infections (Giguère & Afonso, 2013). Reported patterns and trends of resistance to other antimicrobial agents among *S. zooepidemicus* isolates have been less consistent. For instance, while temporal increases in the percentages of antimicrobial- and multidrug-resistant *S. zooepidemicus* isolates were reported in a United Kingdom study (Johns & Adams, 2015), similar trends were not identified in studies conducted in the United States (Erol et al., 2012) and Canada (Malo et al., 2016; Awosile et al., 2018).

*R. equi*, a Gram-positive facultative intracellular coccobacillus, is most commonly associated with pyogranulomatous bronchopneumonia in foals under 6 months of age (Prescott, 1991; Vázquez-Boland et al., 2010). Infection with *R. equi* is typically acquired through inhalation, and the organism is endemic in some breeding farms where it persists in the soil (Takai et al., 1991). Due to its intracellular nature and replication in equine macrophages, combination therapy with rifampin and a macrolide such as erythromycin has been the recommended choice for treatment of *R. equi* bronchopneumonia for several decades (Hillidge, 1987). Resistance of *R. equi* isolates to rifampin and macrolides has largely emerged over the past two decades (Buckley, McManamon & Stanbridge, 2007; Giguère et al., 2010; Burton et al., 2013; Huber et al., 2019), and is of particular concern given the limited drug options for effective treatment of this organism and the high odds of death among foals infected with resistant isolates (Giguère et al., 2010).

Whenever possible, culture and subsequent antimicrobial susceptibility testing are recommended to guide therapy for suspected bacterial infections in order to minimize inappropriate antibiotic use (Morley et al., 2005). In situations where empirical antimicrobial use is necessary in veterinary patients, therapeutic choices should be made based upon suspected pathogen(s) and susceptibility, ideally with timely information that is relevant for the region (Wilson, 2001). Access to current antimicrobial resistance (AMR) surveillance data is therefore essential to enable clinicians to make prudent decisions in these situations. For *S. zooepidemicus,* the most recent laboratory AMR data published in the study area, Kentucky, were collected between 2000 and 2010 (Erol et al., 2012), and therefore dissemination of more up-to-date AMR data is warranted.

Surveillance of antimicrobial co-resistance patterns is also useful, as it can enable the prediction of AMR patterns based on knowledge of resistance to a specific agent. Knowledge of co-resistance may be applied in the clinical setting to inform empirical therapy by suggesting patterns of resistance that may be expected in a patient with previous exposure to specific antimicrobials (Wong et al., 2014). Furthermore, ongoing surveillance of antimicrobial susceptibility patterns and trends among veterinary pathogens is essential to identify isolates with new or emerging resistance, particularly with respect to antimicrobial agents that have human health importance. Indeed, several antimicrobial classes listed by the World Health Organization (WHO) as “critically important” for human health are used in equine practice; these include fluoroquinolones, third and later generation cephalosporins, and macrolides (World Health Organization, 2019).

Given the concerns highlighted above, as well as the substantial clinical impacts of equine respiratory pathogens, the objectives of this study were to investigate and identify (1) antimicrobial resistance patterns and temporal trends among *Streptococcus equi* subsp. *zooepidemicus* and *Rhodococcus equi* isolated from equine specimens submitted to a veterinary diagnostic laboratory in Kentucky between 2012 and 2017, and (2) predictors of antimicrobial and multidrug resistance of the above isolates.

## Methodology

### Data source

Laboratory records were obtained for 5,343 equine clinical specimens submitted to the University of Kentucky Veterinary Diagnostic Laboratory (UKVDL) for isolation and susceptibility testing between January 1, 2012 and December 31, 2017. The following data were extracted for each sample: accession number, sample ID, breed, sex, age, date of submission, county, state, specimen source, bacterial species isolated, and antimicrobial susceptibility test results. Only specimens that were positive for *S*. *zooepidemicus* or *R. equi* were included in the study. *S. zooepidemicus* records were included for analysis if the specimen source was listed as one of the following respiratory tract sites: lung, nasal, tracheal, transtracheal, bronchus, thoracic, thoracic cavity, pharyngeal, throat, and pleura. *R. equi* records from all specimen sites were included for analysis. The data were assessed for duplicate entries and none were found.

### Bacterial isolation

The laboratory that supplied the study data processed submitted samples for bacterial isolation and antimicrobial susceptibility testing. For bacterial isolation, samples were inoculated onto blood agar and eosin methylene blue agar plates, and incubated in 5–10% CO_2_ at 37 °C for 24 h. If the sample was obtained from a site likely to be contaminated, such as nasal mucosa, a Columbia colistin/nalidixic acid (CNA) plate with blood was also inoculated. Plates were examined for bacterial growth, incubated at 37 °C in aerobic incubators for another 24 h, and again examined for growth. Identification of bacterial isolates was made based upon colony morphology, gram staining or dark-field examination, beta-hemolysis, CAMP (Christie, Atkinson, Munch, Peterson) test and standard biochemical test results.

### Antimicrobial susceptibility testing

Antimicrobial susceptibility testing and interpretation were performed using the criteria established by the Clinical and Laboratory Standards Institute (CLSI) (CLSI, 2008; CLSI, 2012; CLSI, 2013a; CLSI, 2013b; CLSI, 2014; CLSI, 2015a; CLSI, 2015b; CLSI, 2016; CLSI, 2017). The following standard strains were tested for quality control for antimicrobial susceptibility testing: *Escherichia coli* ATCC 35218, *Pseudomonas aeruginosa* ATCC 27853, *Streptococcus pneumoniae* ATCC 49619, and *Staphylococcus aureus* ATCC 29213. Isolates were tested for susceptibility to the following antimicrobial agents using broth microdilution: amikacin, ampicillin, azithromycin, cefazolin, ceftazidime, ceftiofur, chloramphenicol, clarithromycin, doxycycline, enrofloxacin, erythromycin, gentamicin, imipenem, oxacillin + 2% NaCl, penicillin, rifampin, tetracycline, ticarcillin, ticarcillin/clavulanic acid, and trimethoprim/ sulfamethoxazole (TMS).

Isolates were classified as susceptible, intermediate, or resistant based upon minimum inhibitory concentration (MIC). For *S. zooepidemicus* isolates, equine-specific interpretive breakpoints were available for the entirety of the study period for ampicillin, ceftiofur, and penicillin (CLSI, 2008; CLSI, 2013a; CLSI, 2015a). Equine-specific breakpoints for cefazolin were published in 2013 (CLSI, 2013a). When veterinary-specific reference breakpoints for interpretation of susceptibility testing did not exist, human criteria were used (CLSI, 2012; CLSI, 2013b; CLSI, 2014; CLSI, 2015b; CLSI, 2016; CLSI, 2017). Minimum inhibitory concentration (in µg/mL) breakpoints for susceptible (S), intermediate (I), and resistant (R) *S. zooepidemicus* isolates were as follows: ampicillin (S: ≤ 0.25), azithromycin (S: ≤ 0.5, I: 1, R: ≥ 2), cefazolin (S: ≤ 8, I: 16, R: ≥ 32; equine breakpoints adopted during 2017: S: ≤ 2, I: 4, R: ≥ 8), ceftazidime (S: ≤ 8, I: 16, R: > 64), ceftiofur (S: ≤ 0.25), chloramphenicol (S: ≤ 4, I: 8, R: ≥ 16), doxycycline (S: ≤ 4, I: 8, R: ≥ 16), enrofloxacin (S: ≤ 0.5, I: 1, R: ≥ 2), erythromycin (S: ≤ 0.25, I: 0.5, R: ≥ 1), imipenem (S: ≤ 4, I: 8, R: ≥ 16), oxacillin (S: ≤ 1, R: ≥ 4), penicillin (S: ≤ 0.12), rifampin (S: ≤ 1, I: 2, R: ≥ 4), tetracycline (S: ≤ 2, I: 4, R: ≥ 8), ticarcillin (S: ≤ 16, I: 32-64, R: > 64), ticarcillin/clavulanic acid (S: ≤ 16/2, I: 32/2-64/2, R: > 64/2), and TMS (S: ≤ 2/38, R: ≥ 4/76). For antimicrobial agents that did not have published MIC breakpoints for β-hemolytic streptococci during the study period (ceftazidime, doxycycline, oxacillin, rifampin, ticarcillin, ticarcillin/clavulanic acid, and TMS), the breakpoints reported above are those that were used by the diagnostic laboratory.

For susceptibility testing of *R. equi* isolates, there are currently no equine-specific interpretive breakpoints approved by the CLSI (CLSI, 2008; CLSI, 2013a; CLSI, 2015a). Therefore, CLSI standards for *R. equi* isolates from humans were followed, which recommend using breakpoints for *S. aureus* (CLSI, 2012; CLSI, 2013b; CLSI, 2014; CLSI, 2015b; CLSI, 2016; CLSI, 2017). Minimum inhibitory concentration breakpoints for susceptible, intermediate, and resistant *R. equi* isolates were as follows: amikacin (S: ≤ 16, I: 32, R: ≥ 64), chloramphenicol (S: ≤ 8, I: 16, R: ≥ 32), clarithromycin (S: ≤ 2, I: 4, R: ≥ 8), doxycycline (S: ≤ 4, I: 8, R: ≥ 16), enrofloxacin (S: ≤ 0.5, I: 1, R: ≥2), erythromycin (S: ≤ 0.5, I: 1-4, R: ≥ 8), gentamicin (S: ≤ 4, I: 8, R: ≥ 16), imipenem (S: ≤ 4, I: 8, R: ≥ 16), rifampin (S: ≤ 1, I: 2, R ≥ 4), tetracycline (S: ≤ 4, I: 8, R: ≥ 16), and TMS (S: ≤ 2/38, R: ≥ 4/76).

The results of antimicrobial susceptibility testing were re-classified as either susceptible or resistant, with isolates listed as “intermediate” or “not susceptible” re-coded as resistant (Magiorakos et al., 2012). In addition, each antimicrobial drug was classified according to the appropriate drug class. Isolates were classified as exhibiting antimicrobial resistance (AMR) if they were resistant to at least one agent from one or more antimicrobial classes, and classified as exhibiting multidrug resistance (MDR) if they were resistant to at least one agent in three or more antimicrobial classes, excluding intrinsic resistance (Magiorakos et al., 2012; Leclercq et al., 2013; Sweeney et al., 2018). Unlike acquired resistance, intrinsic or inherent resistance of bacterial species to antimicrobial classes is not attributable to antibiotic selective pressure, and is excluded from classifications of AMR or MDR (Leclercq et al., 2013; Cox & Wright, 2013). For instance, since *Streptococcus* species exhibit low-level intrinsic resistance to aminoglycosides, these drugs were not assessed for *S. zooepidemicus* resistance (Leclercq et al., 2013). *R. equi*, a facultative intracellular bacterium that replicates within macrophages, has intrinsic resistance to penicillins and cephalosporins, and therefore these antimicrobial classes were not assessed for *R. equi* resistance (Vázquez-Boland et al., 2010; Vázquez-Boland & Meijer, 2019). Azithromycin was also excluded from analysis for *R. equi* because results for all specimens were listed as “no interpretation.”

## Data analysis

### Summary statistics

Statistical analyses were performed using SAS 9.4 (SAS Institute Inc., 2017) and STATA 17.0 (StataCorp, 2021). Separate analyses were performed for *S. zooepidemicus* and *R. equi* isolates. Patient age was assessed for normality of distribution using the Shapiro–Wilk test. As this variable was not normally distributed, median and interquartile range were used for descriptive statistics. Chi-square test (or Fisher’s exact test if appropriate based on sample size) was used to assess for differences of proportions of isolates with respect to the following variables: season and year of submission, patient sex, and breed. Univariable logistic regression was used to assess associations between the above variables and the following outcomes: (a) MDR of *S. zooepidemicus* isolates*,* and (b) resistance to both macrolide(s) and rifampin among *R. equi* isolates. For variables where complete separation of the outcome was present, Firth logistic regression was used to obtain parameter estimates (Firth, 1993; Heinze & Schemper, 2002). Cochran-Armitage tests were used to assess for temporal trends of the above outcomes as well as resistance to each antimicrobial class. Statistical significance for all tests was assessed using a critical *p-*value of ≤ 0.05.

### Associations between drug classes

Multivariable logistic regression models were used to investigate whether resistance of *S. zooepidemicus* and *R. equi* isolates to any of the antimicrobial classes tested could be predicted by their patterns of resistance to other drug classes. For *S. zooepidemicus* models*,* cephalosporins, penicillins, and carbapenems were combined into the category β-lactam antibiotics. Model-building was performed using a two-step process. In the first step, univariable logistic regression was used to assess whether resistance to each antimicrobial class was significantly associated with resistance to any of the other antimicrobial classes. Antimicrobial classes with significant univariable associations at a liberal *p*-value of ≤ 0.15 were then considered as potential predictor variables in multivariable logistic regression models in the second step. Multivariable models were built for both *S. zooepidemicus* and *R. equi*, with resistance to each drug class as outcome variables, using manual backwards elimination and a cutoff *p*-value of 0.05. Variables were considered confounders if their removal resulted in > 20% change in the coefficients for any of the other variables in the model, and were considered for retention in the final models. Hosmer-Lemeshow test was used to assess goodness-of-fit of the final multivariable models.

## Results

### Antimicrobial and multidrug resistance patterns

#### (a) *S. zooepidemicus*

**Figure 1:**
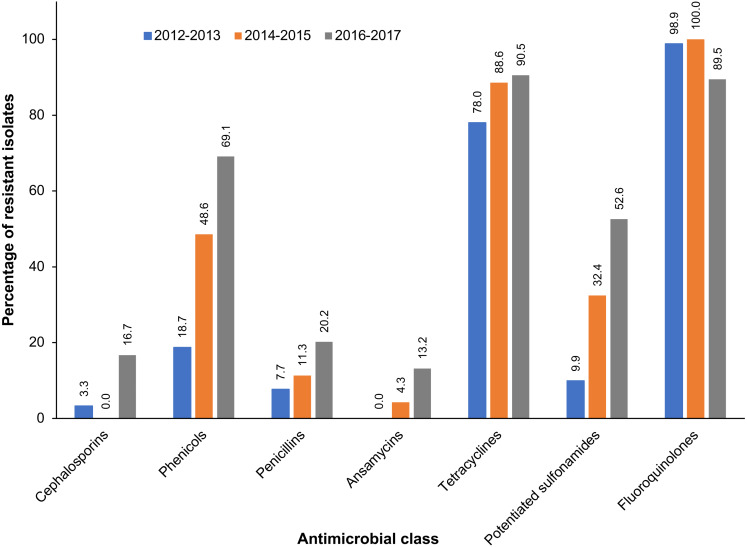
Antimicrobial classes with significant temporal trends of resistance among *S. zooepidemicus* isolated from equine respiratory specimens submitted to a veterinary diagnostic laboratory in Kentucky, USA (2012–2017).

A total of 247 *S. zooepidemicus* isolates were obtained from equine respiratory specimens. Almost all (99.6%) of the *S. zooepidemicus* isolates exhibited resistance to at least one antimicrobial, and 53.3% were multidrug-resistant (Table 1). While there was no significant temporal trend (*p* = 0.222) of AMR, a significant (*p* < 0.001) increase in the proportion of multidrug-resistant *S. zooepidemicus* isolates was observed during the study period. At the beginning of the study period, 25.3% of *S. zooepidemicus* isolates exhibited multidrug resistance, which increased to 73.8% of isolates by the final years of the study. The vast majority of *S. zooepidemicus* isolates exhibited resistance to enrofloxacin (96.2%) and tetracycline (85.3%) (Table 1). In contrast, resistance to penicillin was observed in just 6.9% of *S. zooepidemicus* isolates. Similarly, 6.9% of *S. zooepidemicus* isolates were resistant to one or more cephalosporins, but none exhibited resistance to ceftiofur, a third-generation cephalosporin. Among *S. zooepidemicus* isolates, significant temporal increases in resistance to the following antimicrobial classes were observed during the study period: cephalosporins, phenicols, penicillins, ansamycins, tetracyclines, and potentiated sulfonamides (Table 1, Fig. 1). In contrast, a decreasing trend of resistance was observed for fluoroquinolones. Among multidrug-resistant *S. zooepidemicus* isolates, the most frequently (27.7%) observed pattern of antimicrobial resistance was resistance to chloramphenicol, enrofloxacin, and tetracycline (Table 2).

**Table 1:** Antimicrobial and multidrug resistance among *S. zooepidemicus* isolated from equine respiratory specimens submitted to a veterinary diagnostic laboratory in Kentucky, USA (2012–2017).

	2012–2013	2014–2015	2016–2017	Total	*p*-value, CAT^a^
**AMR** ^b^	100% (91/91)	100% (71/71)	98.8% (83/84)	99.6% (245/246)	0.222
**MDR** ^c^	25.3% (23/91)	64.8% (46/71)	73.8% (62/84)	53.3% (131/246)	< 0.001
**Cephalosporins**	3.3% (3/91)	0% (0/71)	16.7% (14/84)	6.9% (17/246)	0.001
Cefazolin	2.2% (2/91)	0% (0/70)	15.5% (13/84)	6.1% (15/245)	
Ceftazidime	3.3% (3/91)	0% (0/70)	4.0% (3/76)	2.5% (6/237)	
Ceftiofur	0% (0/88)	0% (0/64)	0% (0/62)	0% (0/214)	
**Phenicols**					
Chloramphenicol	18.7% (17/91)	48.6% (34/70)	69.1% (58/84)	44.5% (109/245)	< 0.001
**Fluoroquinolones**					
Enrofloxacin	98.9% (90/91)	100% (71/71)	89.5% (68/76)	96.2% (229/238)	0.002
**Carbapenems**					
Imipenem	0% (0/91)	1.4% (1/70)	3.6% (3/84)	1.6% (4/245)	0.063
**Macrolides**	4.4% (4/91)	7.1% (5/70)	11.9% (10/84)	7.8% (19/245)	0.064
Azithromycin	3.3% (3/91)	4.3% (3/70)	4.8% (4/84)	4.1% (10/245)	
Erythromycin	4.4% (4/91)	5.7% (4/70)	10.7% (9/84)	6.9% (17/245)	
**Penicillins**	7.7% (7/91)	11.3% (8/71)	20.2% (17/84)	13.0% (32/246)	0.014
Ampicillin	4.4% (4/91)	1.4% (1/71)	13.1% (11/84)	6.5% (16/246)	
Oxacillin	2.2% (2/91)	2.9% (2/70)	6.6% (5/76)	3.8% (9/237)	
Penicillin	3.3% (3/91)	8.5% (6/71)	9.5% (8/84)	6.9% (17/246)	
Ticarcillin	1.1% (1/91)	0% (0/70)	6.6% (5/76)	2.5% (6/237)	
Ticarcillin/clavulanate	0% (0/91)	0% (0/70)	10.5% (8/76)	3.4% (8/237)	
**Ansamycins**					
Rifampin	0% (0/90)	4.3% (3/70)	13.2% (10/76)	5.5% (13/236)	< 0.001
**Tetracyclines**	78.0% (71/91)	88.6% (62/70)	90.5% (76/84)	85.3% (209/245)	0.019
Tetracycline	78.0% (71/91)	88.6% (62/70)	90.5% (76/84)	85.3% (209/245)	
Doxycycline	31.9% (29/91)	41.4% (29/70)	27.6% (21/76)	33.3% (79/237)	
**Potentiated sulfonamides**					
TMS^d^	9.9% (9/91)	32.4% (23/71)	52.6% (40/76)	30.3% (72/238)	< 0.001

**Notes.**

a Cochran-Armitage trend test.

b Antimicrobial resistance.

c Multidrug resistance.

d Trimethoprim/sulfamethoxazole.

**Table 2:** Most common patterns of antimicrobial resistance among multidrug-resistant *S. zooepidemicus* and *R. equi* isolated from equine specimens submitted to a veterinary diagnostic laboratory in Kentucky, USA (2012–2017).

Pattern	Number of isolates	Percent
***Streptococcus zooepidemicus***		
CHL-ENR-TET	28	27.7%
CHL-ENR-TET-TMS	19	18.8%
CHL-ENR-TET-DOX	10	9.9%
CHL-ENR-TET-DOX-TMS	6	5.9%
ENR-TET-TMS	6	5.9%
ENR-TET-DOX-TMS	5	5.0%
***Rhodococcus equi***		
CLR-CHL-ENR-ERY-RIF-TET-TMS	10	19.6%
CLR-CHL-ENR-ERY-RIF	9	17.7%
CHL-ENR-TMS	5	9.8%
CLR-ENR-ERY-RIF-TET-TMS	4	7.8%
CHL-ENR-RIF-TET-TMS	3	5.9%
CHL-ENR-TET-TMS	2	3.9%

**Notes.**

CHL chloramphenicol CLR clarithromycin DOX doxycycline ENR enrofloxacin ERY erythromycin RIF rifampin TET tetracycline TMS trimethoprim/sulfamethoxazole

#### (b) *R. equi*

**Figure 2:**
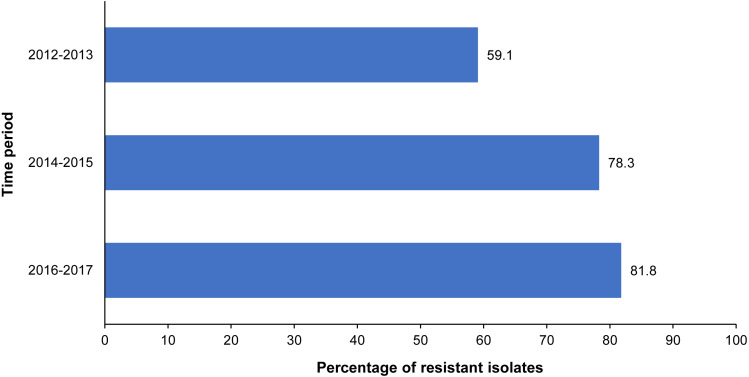
Significant temporal trend of fluoroquinolone resistance among *R. equi* isolated from equine specimens submitted to a veterinary diagnostic laboratory in Kentucky, USA (2012–2017).

There was a total of 182 *Rhodococcus equi* isolates. Overall, 83.0% of the *R. equi* isolates were resistant to at least one antimicrobial, with the proportion of antimicrobial-resistant isolates remaining relatively consistent throughout the study period (Table 3). Just under a quarter (24%) of the *R. equi* isolates were resistant to rifampin. Resistance to at least one macrolide antibiotic was observed in 19.2% of *R. equi* isolates, with 16.5% resistant to clarithromycin and 18.8% to erythromycin. Resistance to both rifampin and at least one macrolide antibiotic was observed in 15.6% of the *R. equi* isolates overall, and a significant temporal trend in the level of resistance to these antimicrobials was not observed (*p* = 0.441). While the proportion of fluoroquinolone-resistant *R. equi* isolates increased significantly during the study period, significant temporal trends were not observed for any other antimicrobial classes (Table 3, Fig. 2). Among multidrug-resistant *R. equi* isolates, the most common antimicrobial resistance pattern included resistance to clarithromycin, chloramphenicol, enrofloxacin, erythromycin, rifampin, tetracycline, and trimethoprim/ sulfamethoxazole, observed in 19.6% of the multidrug-resistant isolates (Table 2).

**Table 3:** Antimicrobial resistance among *R. equi* isolated from equine specimens submitted to a veterinary diagnostic laboratory in Kentucky, USA (2012–2017).

	2012–2013	2014–2015	2016–2017	Total	*p*-value, CAT^a^
**AMR** ^b^	79.5% (35/44)	83.3% (50/60)	84.6% (66/78)	83.0% (151/182)	0.490
**MAC/RIF** ^c^	9.1% (4/44)	20.3% (12/59)	15.8% (12/76)	15.6% (28/179)	0.441
**Aminoglycosides**	4.6% (2/44)	10% (6/60)	3.9% (3/78)	6.0% (11/182)	0.681
Amikacin	2.3% (1/44)	8.3% (5/60)	2.6% (2/78)	4.4% (8/182)	
Gentamicin	2.3% (1/44)	8.3% (5/60)	3.9% (3/78)	5.0% (9/182)	
**Phenicols**					
Chloramphenicol	15.9% (7/44)	33.3% (20/60)	30.8% (24/78)	28.0% (51/182)	0.122
**Fluoroquinolones**					
Enrofloxacin	59.1% (26/44)	78.3% (47/60)	81.8% (63/77)	75.1% (136/181)	0.009
**Carbapenems**					
Imipenem	0% (0/44)	1.7% (1/60)	0% (0/78)	0.6% (1/182)	0.814
**Macrolides**	18.2% (8/44)	21.7% (13/60)	18.0% (14/78)	19.2% (35/182)	0.899
Clarithromycin	13.6% (6/44)	20.0% (12/60)	15.4% (12/78)	16.5% (30/182)	
Erythromycin	15.9% (7/44)	21.7% (13/60)	18.2% (14/77)	18.8% (34/181)	
**Ansamycins**					
Rifampin	18.2% (8/44)	30.5% (18/59)	22.4% (17/76)	24.0% (43/179)	0.774
**Tetracyclines**	20.5% (9/44)	26.7% (16/60)	12.8% (10/78)	19.2% (35/182)	0.191
Doxycycline	2.3% (1/44)	3.3% (2/60)	5.1% (4/78)	3.9% (7/182)	
Tetracycline	18.2% (8/44)	27.1% (16/59)	10.4% (8/77)	17.8% (32/180)	
**Potentiated sulfonamides**					
TMS^d^	34.1% (15/44)	44.8% (26/58)	21.8% (17/78)	32.2% (58/180)	0.075

**Notes.**

a Cochran-Armitage trend test.

b Antimicrobial resistance.

c Resistant to ansamycins (rifampin) and one or more macrolide antibiotics.

d Trimethoprim/sulfamethoxazole.

### Patient characteristics & predictors of antimicrobial resistance

#### (a) *S. zooepidemicus*

Median age of animals whose samples were positive for *S. zooepidemicus* was 52 weeks, and ranged from 3 h to 24 years, with an interquartile range of eight weeks to four years. There was an even distribution of samples by sex, with 50.3% (86/171) from females and 49.7% (85/171) from males. The distribution of the *S. zooepidemicus-*positive samples by breed was as follows: 70.1% of specimens (157/224) were obtained from Thoroughbreds, 7.1% (16/224) from Quarter Horses, and 6.7% (15/224) from Saddlebreds. Other breeds included: Tennessee Walking Horse, Warmblood, Rocky Mountain Horse, Standardbred, Arabian, Draft, Pony, and mixed breed. There was a significant (*p* = 0.018) difference in the percentage of *S. zooepidemicus* samples submitted by season, with spring having the highest (33.6%), followed by summer (23.1%), winter (22.7%), and the fewest submitted during the fall (20.7%).

Among *S. zooepidemicus* isolates*,* year of submission was the only significant (*p* < 0.001) predictor of MDR (Table 4). The odds of MDR among *S. zooepidemicus* isolates from specimens submitted between 2014 and 2015 were 5.4 times those of MDR among isolates submitted between 2012 and 2013 (95% CI [2.8–10.7]). Isolates from specimens submitted between 2016 and 2017 had even higher odds of MDR compared to isolates submitted at the beginning of the study period (OR = 8.3; 95% CI [4.3–16.4]). None of the other categorical variables had significant univariable associations with MDR among *S. zooepidemicus* isolates (Table 4). Similarly, patient age was not a significant predictor of MDR (*p* = 0.430).

**Table 4:** Distribution and univariable associations of potential explanatory variables with MDR among *S. zooepidemicus* and macrolide/rifampin resistance among *R. equi* isolated from equine specimens submitted to a veterinary diagnostic laboratory in Kentucky, USA (2012–2017).

Variable	Multidrug-resistant *S. zooepidemicus*		Macrolide/rifampin resistant *R. equi*	
	% (n/N)	OR^a^ (95% CI^b^)	% (n/N)	OR^a^ (95% CI^b^)
**Season**		*p* = 0.667		*p* = 0.136
Spring	50.6% (42/83)	ref.	7.9% (5/63)	ref.
Winter	54.6% (30/55)	1.2 (0.6, 2.3)	0% (0/7)	0.7 (0.03, 17.1)
Summer	59.7% (34/57)	1.4 (0.7, 2.9)	20.2% (19/94)	2.7 (1.0, 7.6)
Fall	49.0% (25/51)	0.9 (0.5, 1.9)	26.7% (4/15)	4.2 (1.0, 17.4)
**Year**		*p* < 0.001		*p* = 0.312
2012–2013	25.2% (23/91)	ref.	9.1% (4/44)	ref.
2014–2015	64.8% (46/71)	5.4 (2.8, 10.7)	33.0% (12/59)	2.6 (0.8, 8.5)
2016–2017	73.8% (62/84)	8.3 (4.2, 16.4)	15.8% (12/76)	1.9 (0.6, 6.2)
**Breed**		*p* = 0.576		*p* = 0.108
Thoroughbred	56.4% (88/156)	ref.	19.1% (28/147)	ref.
Other	44.4% (16/36)	0.6 (0.3, 1.3)	0% (0/22)	0.1 (0.005, 1.6)
Quarter Horse	50.0% (8/16)	0.8 (0.3, 2.2)	–	–
Saddlebred	60.0% (9/15)	1.2 (0.4, 3.4)	–	–
**Sex**		*p* = 0.928		*p* = 0.662
Female	58.1% (50/86)	ref.	15.6% (10/64)	ref.
Male	58.8% (50/85)	1.0 (0.6, 1.9)	18.4% (14/76)	1.2 (0.5, 3.0)
**Age** (weeks)	–	*p* = 0.430	–	*p* = 0.557
		1.0 (0.9, 1.0)		1.0 (0.9, 1.0)

**Notes.**

a Odds ratio.

b Confidence interval.

#### (b) *R. equi*

Information on sampling site was available for 168 *R. equi-*positive specimens. The most frequent site of sample collection was the lung (55.4%; 93/168), followed by abscesses (20.2%; 34/168) and lymph nodes (5.4%; 9/168). Other sampling sites included joint (6 isolates), liver (6), trachea (8), abdomen (3), colon (2), placenta (2), bone (1), mesentery (1), nose (1), spinal cord (1), and vertebral column (1). Sampling site was missing for 14 *R. equi-*positive specimens. The ages of animals with *R. equi-*positive specimens ranged from 12 h to 23 years, with a median age of 8.6 weeks, and an interquartile range of 8 to 14 weeks. Among *R. equi-*positive specimens, 45.8% (65/142) were obtained from females, and 54.2% (77/142) were from males. The majority (87.2%, 150/172) of the *R. equi-*positive specimens were obtained from Thoroughbreds. Other breeds included: American Saddlebred, Tennessee Walking Horse, Quarter Horse, Rocky Mountain Horse, Standardbred, Morgan, Warmblood, Pony, and mixed breed. There was a significant (*p* < 0.001) difference in the proportion of *R. equi*-positive samples submitted by season. Most specimens were either submitted during the summer (52.2%) or spring (35.2%), and the fewest samples were submitted during the fall (8.8%) and winter (3.9%) months. Patient characteristics, season and year of submission were not significantly associated with resistance to macrolide(s) and rifampin among *R. equi* isolates (Table 4).

### Does resistance to one antimicrobial class predict resistance to other drug classes?

#### (a) *S. zooepidemicus*

Results of multivariable logistic regression models indicated that for *S. zooepidemicus*, resistance of an isolate to one antimicrobial class could predict its resistance to other drug classes (Table 5). Significant predictors of *β*-lactam resistance included resistance to macrolides (OR = 14.7; 95% CI [4.6–46.8]; *p* < 0.001), ansamycins (OR = 9.3; 95% CI [2.2–40.0]; *p* = 0.003) and fluoroquinolones (OR = 0.13; 95% CI [0.03–0.66]; *p* = 0.014). Resistance to macrolides (OR = 3.2; 95% CI [1.2–8.7]; *p* = 0.023) and phenicols (OR = 3.4; 95% CI [1.9–6.3]; *p* < 0.001) were associated with higher odds of resistance to potentiated sulfonamides. Resistance to β-lactams (OR = 10.1; 95% CI [2.4–42.2]; *p* = 0.002) and phenicols (OR = 15.3; 95% CI [1.8–131.2]; *p* = 0.013) were significant predictors of ansamycin resistance. However, macrolide resistance was a confounder in the association between β-lactam resistance and ansamycin resistance, and therefore this variable was retained in the final ansamycin model. Resistance to tetracyclines (OR = 4.0; 95% CI [1.6–10.3]; *p* = 0.004), potentiated sulfonamides (OR = 2.8; 95% CI [1.5–5.1]; *p* = 0.001), and ansamycins (OR = 9.4; 95% CI [1.2–75.8]; *p* = 0.035) were significant predictors of resistance to phenicols. In the models with outcomes of tetracycline resistance, macrolide resistance, and fluoroquinolone resistance, only univariable associations were observed (Table 5).

**Table 5:** Predictors of antimicrobial resistance to different drug classes among *S. zooepidemicus* isolated from equine respiratory specimens submitted to a veterinary diagnostic laboratory in Kentucky, USA (2012–2017).

Dependent variable	Predictor		OR^a^	95% CI^b^	*p*-value	Hosmer-Lemeshow GOF^c^ test *p-*value
β-lactams	Macrolides	Resistant	14.7	4.6, 46.8	< 0.001	0.594
		Susceptible	Referent	–	–	
	Ansamycins	Resistant	9.3	2.2, 40.0	0.003	
		Susceptible	Referent	–	–	
	Fluoroquinolones	Resistant	0.13	0.03, 0.66	0.014	
		Susceptible	Referent	–	–	
Tetracyclines	Phenicols	Resistant	4.9	1.9, 12.2	< 0.001	–
		Susceptible	Referent	–	–	
Macrolides	β-lactams	Resistant	14.7	5.2, 41.8	< 0.001	–
		Susceptible	Referent	–	–	
Potentiated sulfonamides	Macrolides	Resistant	3.2	1.2, 8.7	0.023	0.963
		Susceptible	Referent	–	–	
	Phenicols	Resistant	3.4	1.9, 6.3	< 0.001	
		Susceptible	Referent	–	–	
Ansamycins	β-lactams	Resistant	10.1	2.4, 42.2	0.002	0.201
		Susceptible	Referent	–	–	
	Macrolides	Resistant	4.2	0.9, 19.2	0.066	
		Susceptible	Referent			
	Phenicols	Resistant	15.3	1.8, 131.2	0.013	
		Susceptible	Referent	–	–	
Phenicols	Tetracyclines	Resistant	4.0	1.6, 10.3	0.004	0.978
		Susceptible	Referent	–	–	
	Potentiated sulfonamides	Resistant	2.8	1.5, 5.1	0.001	
		Susceptible	Referent	–	–	
	Ansamycins	Resistant	9.4	1.2, 75.8	0.035	
		Susceptible	Referent	–	–	
Fluoroquinolones	β-lactams	Resistant	0.12	0.03, 0.46	0.002	–
		Susceptible	Referent	–	–	

**Notes.**

a Odds ratio.

b Confidence interval.

c Goodness-of-fit.

#### (b) *R. equi*

Significant associations between antimicrobial resistance to different drug classes were also observed among *R. equi* isolates (Table 6). For instance, significant predictors of macrolide resistance included resistance to phenicols (OR = 3.7; 95% CI [1.3–10.6]; *p* = 0.013) and ansamycins (OR = 19.9; 95% CI [6.9–56.9]; *p* < 0.001). Similarly, resistance to macrolides (OR = 3.7; 95% CI [1.2–11.3]; *p* = 0.020) and ansamycins (OR = 5.5; 95% CI [2.0–14.9]; *p* < 0.001), along with fluoroquinolone resistance (OR = 5.3; 95% CI [1.4–19.4]; *p* = 0.012), were predictors of resistance to phenicols. Significant predictors of tetracycline resistance were resistance to aminoglycosides (OR = 12.4; 95% CI [1.9–81.3]; *p* = 0.009), ansamycins (OR = 12.1; 95% CI [4.3–34.2]; *p* < 0.001), and potentiated sulfonamides (OR = 13.3; 95% CI [4.5–39.9]; *p* < 0.001). Finally, significant predictors of ansamycin resistance were resistance to macrolides (OR = 15.6; 95% CI [5.2–46.5]; *p* < 0.001), phenicols (OR = 4.6; 95% CI [1.7–12.8]; *p* = 0.003), and tetracyclines (OR = 5.6; 95% CI [1.9–16.5]; *p* = 0.002).

**Table 6:** Predictors of antimicrobial resistance to different drug classes among *R. equi* isolated from equine specimens submitted to a veterinary diagnostic laboratory in Kentucky, USA (2012–2017).

Dependent variable	Predictor		OR^a^	95% CI^b^	*p*-value	Hosmer–Lemeshow GOF^c^ test *p*-value
Aminoglycosides	Tetracyclines	Resistant	8.9	2.5, 32.6	< 0.001	–
		Susceptible	Referent	–	–	
Macrolides	Phenicols	Resistant	3.7	1.3, 10.6	0.013	0.196
		Susceptible	Referent	–	–	
	Ansamycins	Resistant	19.9	6.9, 56.9	< 0.001	
		Susceptible	Referent	–	–	
Phenicols	Macrolides	Resistant	3.7	1.2, 11.3	0.020	0.725
		Susceptible	Referent	–	–	
	Fluoroquinolones	Resistant	5.3	1.4, 19.4	0.012	
		Susceptible	Referent	–	–	
	Ansamycins	Resistant	5.5	2.0, 14.9	< 0.001	
		Susceptible	Referent	–	–	
Tetracyclines	Aminoglycosides	Resistant	12.4	1.9, 81.3	0.009	0.081
		Susceptible	Referent	–	–	
	Ansamycins	Resistant	12.1	4.3, 34.2	< 0.001	
		Susceptible	Referent	–	–	
	Potentiated sulfonamides	Resistant	13.3	4.5, 39.9	< 0.001	
		Susceptible	Referent	–	–	
Fluoroquinolones	Phenicols	Resistant	7.6	2.2, 25.9	0.001	–
		Susceptible	Referent	–	–	
Ansamycins	Macrolides	Resistant	15.6	5.2, 46.5	< 0.001	0.246
		Susceptible	Referent	–	–	
	Phenicols	Resistant	4.6	1.7, 12.8	0.003	
		Susceptible	Referent	–	–	
	Tetracyclines	Resistant	5.6	1.9, 16.5	0.002	
		Susceptible	Referent	–	–	
Potentiated sulfonamides	Tetracyclines	Resistant	10.2	4.3, 24.0	< 0.001	–
		Susceptible	Referent	–	–	

**Notes.**

a Odds ratio.

b Confidence interval.

c Goodness-of-fit.

## Discussion

This retrospective study investigated patterns and predictors of antimicrobial resistance among isolates of two bacterial pathogens often implicated in respiratory infections in horses, *Rhodococcus equi* and *Streptococcus equi* subsp. *zooepidemicus*, from clinical specimens submitted to the University of Kentucky Veterinary Diagnostic Laboratory (UKVDL) between 2012 and 2017.

### Antimicrobial resistance and MDR

#### (a) *S. zooepidemicus* isolates

Penicillins are recommended as the first-line treatment for suspected *S. zooepidemicus* infections because in general, penicillin resistance among *S. zooepidemicus* has remained low (Giguère & Afonso, 2013). While the level of penicillin resistance in the present study (6.9%) was comparable to reports from Canada (5%) and the United Kingdom (4.5%) (Clark et al., 2008; Johns & Adams, 2015), others have reported minimal to no penicillin resistance among *S. zooepidemicus* isolates (Erol et al., 2012; Van Spijk et al., 2016; Malo et al., 2016; Awosile et al., 2018). The temporal increase observed in this study also contrasts with the findings of previous reports (Johns & Adams, 2015; Malo et al., 2016; Awosile et al., 2018). Nonetheless, the majority of *S. zooepidemicus* isolates in this study population appear to remain susceptible to penicillin, supporting its continued use as a first-line treatment for suspected *S. zooepidemicus* infections. However, the observed trend suggests that the susceptibility profile of *S. zooepidemicus* may be less predictable than previously reported (Giguère & Afonso, 2013), highlighting the importance of culture and susceptibility for guiding antimicrobial therapy when possible.

The increase in cephalosporin resistance among *S. zooepidemicus* isolates in the present study was largely accounted for by resistance to the first-generation cefazolin, while there was minimal to no resistance to third-generation cephalosporins, consistent with previous reports (Clark et al., 2008; Johns & Adams, 2015; Van Spijk et al., 2016; Malo et al., 2016; Awosile et al., 2018). From a public health standpoint, the WHO has deemed third- and later generation cephalosporins as critically important for human medicine (World Health Organization, 2019). Given that penicillin appears to be effective against most *S. zooepidemicus* isolates, and the potential for cephalosporin use in horses to promote multidrug resistance in commensal fecal *E. coli* (Dunowska et al., 2006), cephalosporins should only be used to treat *S. zooepidemicus* infections when deemed necessary based on culture and susceptibility testing.

While the majority of *S. zooepidemicus* isolates were susceptible to β-lactam antibiotics, a concerning finding of the present study was the substantial levels of resistance to other antimicrobials that are important in equine practice. For instance, resistance to trimethoprim/sulfamethoxazole (TMS), a commonly used antimicrobial combination, exhibited a significant temporal trend, increasing from 9.9% to 52.6%. Reported resistance to TMS has varied widely among *S. zooepidemicus*, ranging from 5.7% to as high as 83.5% (Clark et al., 2008; Erol et al., 2012; Johns & Adams, 2015; Van Spijk et al., 2016; Malo et al., 2016; Awosile et al., 2018). Furthermore, the vast majority of isolates (85.3% overall) were resistant to tetracycline, and almost half (44.5%) were resistant to chloramphenicol, both of which had significant temporal increases.

Macrolide resistance among *S. zooepidemicus* isolates was mostly accounted for by resistance to erythromycin (6.9%). Among samples submitted to UKVDL between 2000 and 2010, erythromycin resistance was even less frequent (2.2%), suggesting a temporal increase in macrolide resistance over a longer-term period, despite the lack of a significant trend during this study (*p* = 0.064) (Erol et al., 2012). Resistance to rifampin among *S. zooepidemicus* isolates did increase significantly, from 0% to 13.2%. Neither rifampin nor macrolides are recommended for first-line treatment of *S. zooepidemicus* infections, but these drugs are frequently used to treat *R. equi* infections (Giguère & Afonso, 2013). Antibiotic exposure of commensal *S. zooepidemicus* during treatment of other infections may be related to the emergence of resistance in clinical isolates from respiratory infections.

Over the course of the study period, the temporal increases in resistance of *S. zooepidemicus* to several classes of antimicrobials were reflected in a substantial increase in the level of MDR. Between 2016 and 2017, the percentage of multidrug-resistant isolates reached 73.8%, much higher than previously reported in studies from the U.K. (25.8%) and Atlantic Canada (1.1%) (Johns & Adams, 2015; Awosile et al., 2018). Differences between the present study and previous reports with respect to multidrug resistance may reflect differing patterns of antimicrobial use, as the studies were conducted in various geographic locations and spanned different time periods.

#### (a) *R. equi isolates*

The present study did not identify temporal trends of resistance to rifampin, macrolides, or the combination of the two among *R. equi* isolates, in contrast to previous reports of increasing temporal trends (Buckley, McManamon & Stanbridge, 2007; Giguère et al., 2010; Huber et al., 2019). The absence of such temporal changes in the present study could be attributable to alterations in antimicrobial use protocols due to increased awareness of emerging macrolide and rifampin resistance, but prior antimicrobial use data were not available to assess this in the current study. Regardless, the observed levels of resistance to macrolides (19.2%), rifampin (24%), and the combination of these agents (15.6%) were substantial given the limited number of effective antimicrobials for treatment of *R. equi* infections. Continued monitoring of susceptibility patterns in the region is warranted to determine whether the proportion of isolates with this resistance profile has truly reached a plateau or will continue to rise.

Among the antimicrobial drugs analyzed in this study, enrofloxacin had the highest proportion of resistant isolates. While enrofloxacin has been previously reported to be highly effective against *R. equi in vitro* (Carlson et al., 2010), 75.1% of the isolates in the current study were resistant to enrofloxacin, and an increasing temporal trend in proportion of enrofloxacin-resistant *R. equi* was observed.

A single isolate was resistant to imipenem (0.6%, 1/182), consistent with findings from other studies that have reported high levels of *in vitro* activity of imipenem against *R. equi* isolates (Nordmam & Ronco, 1992; Jacks, Giguère & Nguyen, 2003; Giguère et al., 2010; Riesenberg et al., 2013). Similarly, the majority of *R. equi* isolates were susceptible to aminoglycosides, with only 4.4% showing resistance to amikacin and 5.0% to gentamicin. This is consistent with previous reports of effective *in vitro* activity of aminoglycosides against *R. equi* (Nordmam & Ronco, 1992; Jacks, Giguère & Nguyen, 2003; Carlson et al., 2010; Giguère et al., 2010; Berghaus et al., 2015). However, the reported *in vitro* efficacy of aminoglycosides has not corresponded with favorable *in vivo* outcomes, which has been attributed to lipid insolubility and poor penetration of macrophages (Sweeney, Sweeney & Divers, 1987).

### Does resistance to one antimicrobial class predict resistance to other drug classes?

#### (a) *S. zooepidemicus isolates*

Findings of the current study indicate that resistance of *S. zooepidemicus* isolates to one antimicrobial class can be predicted by resistance to other antimicrobials. Further research to identify antibiotic resistance mechanisms among *S. zooepidemicus* isolates will be valuable for understanding the cause of the observed associations. For example, resistance to phenicols was a significant predictor of tetracycline resistance, and vice versa. In addition, combined chloramphenicol-tetracycline resistance was observed in the majority of multidrug-resistant *S. zooepidemicus* isolates. These associations may be consistent with co-transferrance of the genes conferring resistance to these agents, which occurs in other *Streptococcus* species (Clewell & Gawron-Burke, 1986; Roberts & Schwarz, 2016).

β-lactam resistance was a significant predictor of ansamycin resistance among *S. zooepidemicus* isolates, but macrolide resistance was a confounder in this relationship. This likely reflects the frequent use of macrolide antibiotics in combination with rifampin (Hillidge, 1987; Giguère & Afonso, 2013), as rifampin monotherapy is not recommended (Takai et al., 1997). Combined macrolide-rifampin treatment in foals with subclinical pneumonia has been shown to increase antimicrobial resistance genes for multiple drug classes among fecal bacteria (Álvarez Narváez et al., 2020). Further research is warranted to characterize patterns and predictors of antimicrobial and multidrug resistance among commensal organisms of the equine pharynx, including *S. zooepidemicus*, and to determine how this relates to antimicrobial resistance in pathogens isolated from horses with clinical respiratory tract infections.

#### (b) *R. equi isolates*

As with *S. zooepidemicus,* AMR among *R. equi* isolates could be predicted by resistance to other drug classes. Significant associations between macrolide and rifampin resistance were identified, consistent with the findings of previous reports indicating that many macrolide-resistant *R. equi* isolates are also resistant to rifampin (Carlson et al., 2010; Giguère et al., 2010; Anastasi et al., 2015; Huber et al., 2019). In addition, along with ansamycin (rifampin) resistance, resistance to phenicols was a significant predictor of macrolide resistance. This finding is consistent with those of Carlson and colleagues, who reported significantly higher minimum inhibitory concentrations (MICs) for chloramphenicol and rifampin among macrolide-resistant *R. equi* isolates compared to macrolide-susceptible isolates (Carlson et al., 2010). Furthermore, that study reported that enrofloxacin and gentamicin activity did not significantly differ based upon macrolide susceptibility, consistent with our findings that these antimicrobials were not significant predictors of macrolide resistance (Carlson et al., 2010).

### Strengths and limitations

A key methodological strength of this study is the use of Firth models in situations when the ordinary logistic models could not fit the data. One limitation is the small sample size for some of the sub-analyses, resulting in broad confidence intervals in the final logistic regression models. In addition, medical history (including hospitalization status, clinical setting, antimicrobial use, disease status, sampling method, and outcome) was not available. Therefore, these clinical factors could not be investigated as potential predictors of AMR and MDR. Finally, equine-specific MIC breakpoints are not available for some of the antimicrobial agents reported for *S. zooepidemicus,* or for any antimicrobial agents for *R. equi*. Despite these limitations, the results of the study provide a useful indication of the burden and temporal trends of antimicrobial resistance among bacterial isolates commonly implicated in equine respiratory infections. These findings are particularly relevant for informing clinical decisions of equine practitioners in the region.

## Conclusions

This study identified increasing temporal trends in resistance to several antimicrobial classes as well as MDR among *S. zooepidemicus* isolates, which may be indicative of increasing selection pressure due to antimicrobial use. For several drug classes, the proportion of resistant *S. zooepidemicus* isolates was higher than previously reported. However, the findings of this study indicate that while a substantial proportion of *R. equi* isolates were resistant to macrolides and rifampin, resistance to these drugs did not increase significantly during the study period. In addition, findings of the study indicate that resistance of an isolate to one class of antimicrobials can be used to predict potential resistance to other drug classes, knowledge that may be applied to guide clinical decisions. Continued research assessing these associations among clinical isolates in the region, as well as in other geographic areas, is warranted to evaluate the external validity of these predictions. The findings of this study highlight the importance of continuing to monitor susceptibility patterns of clinically important pathogens in order to identify temporal trends, emerging resistance profiles, and directions for future research.

##  Supplemental Information

10.7717/peerj.13682/supp-1 Supplemental Information 1 Study data for Rhodococcus

10.7717/peerj.13682/supp-2 Supplemental Information 2 Study Data for Streptococcus

## Additional Information and Declarations

### Data Availability

The following information was supplied regarding data availability: The raw study data are available in the Supplementary Files.
